# Impact of ultra-low temperature storage on serum sIgE detection and allergic disease biobank feasibility

**DOI:** 10.1038/s41598-023-47915-x

**Published:** 2023-11-27

**Authors:** Zhenglin Chang, Haisheng Hu, Xiaocong Pan, Changlian Liu, Kemin Liu, Yanxi Zhang, Shiliang Xu, Jiahao Cheng, Qitai Zhang, Qiongqiong Wan, Lexin Xiao, Xueqing Liang, Huimin Huang, Zhangkai J. Cheng, Baoqing Sun

**Affiliations:** Department of Clinical Laboratory, National Center for Respiratory Medicine, National Clinical Research Center for Respiratory Disease, State Key Laboratory of Respiratory Disease, Guangzhou Institute of Respiratory Health, The First Affiliated Hospital of Guangzhou Medical University, Guangzhou, China

**Keywords:** Immunochemistry, Proteins

## Abstract

Research has shown that the concentration and composition of biological samples may change after long-term ultra-low temperature storage. Consequently, this study examined the effect of ultra-low temperature storage on serum sIgE detection by comparing sIgE concentrations at various durations from the time of sample storage to subsequent testing. We selected 40 serum samples from the Guangzhou Medical University Affiliated First Hospital Biobank, which had been tested for house dust mites, dog hair, tobacco mold, cockroaches, and cow milk allergen sIgE. Samples were categorized by storage duration: 14 samples stored for 10 years, 12 for 5 years, and 14 for 3 years. They were also classified by sIgE positive levels: 15 samples at levels 1–2, 15 at levels 3–4, and 10 at levels 5–6. The allergen sIgE of these samples was retested using the same technology. Regardless of the type of allergen or the level of positivity, the majority of sIgE concentrations measured at the time of storage were higher than the current measurements, but the difference was not statistically significant. The correlation between the sIgE results at the time of storage and the current results was high for samples stored for 10 years (rs = 0.991, P < 0.001) and 5 years (rs = 0.964, P < 0.001). Serum allergen sIgE is stable when stored under ultra-low temperature conditions, making the construction of a biological sample bank for allergic diseases feasible. This will facilitate researchers in quickly obtaining samples, conducting technical research, and translating findings, thereby promoting the development of the field of allergy through integration of industry, academia, and research.

## Introduction

Allergy, one of the most common chronic conditions globally, is an important public health concern due to significant quality-of-life and economic impact^1–3^. In recent years, the study of allergic diseases has gained significant attention due to the increasing prevalence of allergies worldwide^4–7^. Immunoglobulin E (IgE)-mediated allergy plays a role in the inflammatory processes of various allergic respiratory diseases, affecting approximately one-third of the world's population^8^. Recent research reveals that with the capability to detect IgE levels down to 0.1 KUA/L, maintaining stable IgE concentration during sample storage is crucial to prevent result discrepancies^9^. The precise detection and analysis of IgE in serum samples are vital for the diagnosis of allergies, comprehension of the underlying mechanisms of allergic reactions, and the advancement of targeted therapeutic strategies^10,11^.

BioBanks have assumed a central role in the era of precision medicine, with the availability of extensive collections of patient samples, complete with well-annotated clinical and pathological data, becoming a critical component for personalized care^12–15^. Ultra-low temperature storage, an indispensable technology in BioBanks, facilitates the preservation of frozen serum or plasma, commonly utilized in retrospective studies^16^. However, concerns arise regarding the long-term storage of biological samples at such temperatures, as potential changes in concentration and composition may compromise the reliability of specific detections, such as sIgE, thereby posing challenges to ongoing research and diagnostic accuracy^17–19^. For instance, donkey serum has been demonstrated to remain stable when stored at − 80 °C for up to a year^16^. However, the concentration of calreticulin protein shows a significant shift after being stored at − 80 °C for more than 8 weeks, with a marked increase observed after 16 weeks^20^. Ferritin showcases impressive stability, with a consistent shelf life of 3–5 years when stored at − 80 °C^21^. Additionally, despite multiple freeze–thaw cycles, serum samples at − 80 °C display only minimal decreases in absolute CK levels^22^. Therefore, the impact of ultra-low-temperature storage varies for different proteins. While some studies have shown that sIgE to allergens in serum can be stored stably at − 20 °C for 17 days^23^, current research on the stability of serum sIgE to allergens under ultra-low temperature storage for a long time is still lacking.

Therefore, this study embarked on a novel exploration by selecting serum samples stored at ultra-low temperatures to analyze the effect of such storage on serum sIgE detection. By comparing sIgE concentrations at the time of sample storage with subsequent testing, the research assessed the feasibility of employing an allergic disease BioBank for insights into allergen distribution, allergic disease pathogenesis, and the refinement of diagnostic techniques. This pioneering work not only contributes to our understanding of ultra-low temperature storage's impact on specific proteins but also offers valuable guidance for the construction of an allergic disease BioBank and the advancement of related storage technologies.

## Methods

### General information

All experimental protocols were consistent with guidelines approved by the Ethical/Institutional Review Board at the First Affiliated Hospital of Guangzhou Medical University (China). 40 serum samples from the Guangzhou Medical University Affiliated First Hospital Biobank, which had been previously tested for house dust mites (d1), dog hair (e5), tobacco mold (m3), cockroaches (i6), and cow milk (f2) allergen sIgE using the Phadia 1000 assay were included in the study. The group consisted of 30 females (75.0%) with a mean age of 21.18 ± 17.63 years. Samples were divided into groups based on their length of storage (14 samples stored for 10 years, 12 samples stored for 5 years, and 14 samples stored for 3 years) and positive sIgE levels (15 samples of level 0–2, 15 samples of level 3–4, and 10 samples of level 5–6).

### Freezing storage condition

The serum samples were stored in an ultra-low temperature freezer at − 80 °C (Haier, DW-86L829BPT). The process is as follows: After clinical testing, the serum samples were aliquoted into 500 μL cryovials. These vials were then systematically placed into paper-based storage boxes with a 10 × 10 grid. Subsequently, the boxes were placed on metal storage racks inside the storage container for long-term preservation. No freezing additives were used in this process.

### Detection methods

4 ml of each serum sample was taken, and after centrifuging at 3000 rpm for 10 min, sIgE concentrations of d1, e5, m3, i6, and f2 allergens in the patient's serum were detected using the Phadia 1000 Fluorescence Enzyme Immunoassay (ImmunoCAP) system (Thermo Fisher, Sweden), following instructions. The detection result was considered positive if it exceeded 0.35 kU/L. The sIgE result was categorized into levels 1–6 based on the radioallergosorbent test (RAST): level 1 0.35–0.7 kU/L, level 2 0.7–3.5 kU/L, level 3 3.5–17.5 kU/L, level 4 17.50–50 kU/L, level 50–100 kU/L, and level 6 ≥ 100.00 kU/L.

### Statistical methods

Data were analyzed using SPSS 25.0 software. Non-normally distributed quantitative data were expressed as median (25%, 75%), and differences between groups were analyzed using the non-parametric Wilcoxon test for paired samples. Differences between three or more groups were analyzed using Wilcoxon signed-rank tests. Spearman correlation analyses were used to analyze the correlation between sIgE results at the time of storage and in subsequent testing. Kappa values were used to evaluate the consistency between the two levels. P < 0.05 indicated statistically significant differences.

## Results

### Differences between samples at the time of storage and in subsequent testing for sIgE allergens

**Figure 1:**
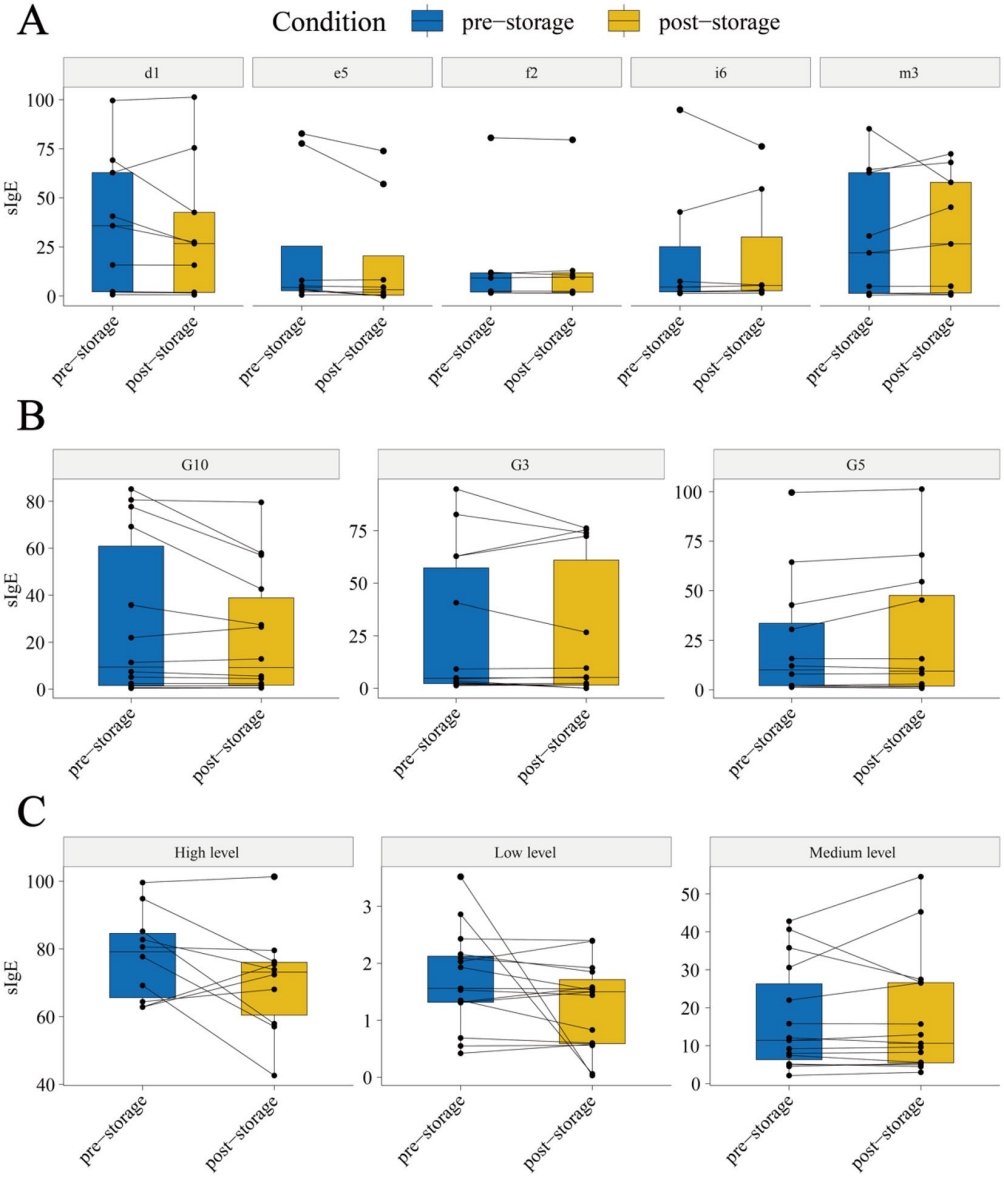
Paired boxplot of serum levels of various allergens: comparison between sige before and after storage, across different storage times and positivity levels. d1: House dust mite; e5: Dog hair; m3: Mold; i6: Cockroach; f2: Milk; G3: samples stored for 3 years; G5: samples stored for 5 years; G10: samples stored for 10 years; High level: samples of level 0–2; Medium level: level 3–4; Low level: level 5–6.

As shown in Table 1 and Fig. 1A, the sIgE level of house dust mite allergen [35.8 kU/L (2.16, 62.84)] as well as dog hair [4.36 kU/L (2.66, 25.40)], cockroach [4.55 kU/L (2.09, 25.12)], and cow milk [9.20 kU/L (1.20, 11.75)] allergens at the time of storage was higher than in subsequent testing [26.69 kU/L (1.85, 42.6), 3.21 kU/L (0.44, 20.46), 5.34 kU/L (2.70, 30.06), and 9.63 kU/L (1.98, 11.77)], but the difference was not statistically significant (P > 0.05). Among them, only the sIgE level of tobacco mold allergen at the time of storage [22.00 kU/L (1.35, 62.83)] was lower than in subsequent testing [26.53 kU/L (1.58, 57.91)] (P > 0.05). There was one sample of house dust mite allergen that changed from a high positive level to a medium positive level, one sample of dog hair allergen that changed from a medium positive level to a low positive level, and one sample of cockroach allergen that changed from a medium positive level to a high positive level (Table S1). The correlation between sIgE levels at the time of storage and in subsequent testing was strongest for cockroach allergens (*r*_*s*_ = 1.000, P < 0.001), followed by house dust mite allergens (*r*_*s*_ = 0.967, P < 0.001, Table 2). The consistency was highest for tobacco mold allergens and cow milk allergens (Kappa = 1.000), followed by house dust mite allergens (Kappa = 0.862, Table 2).

**Table 1:** Difference between serum levels of different allergens upon sample entry and sIgE measured.

sIgE	Pre-storage (kU/L)	Post-storage (kU/L)	P
d1	35.8 (2.16, 62.84)	26.69 (1.85, 42.6)	0.72
e5	4.36 (2.66, 25.40)	3.21 (0.44, 20.46)	0.43
m3	22.00 (1.35, 62.83)	26.53 (1.58, 57.91)	0.92
i6	4.55 (2.09, 25.12)	5.34 (2.70, 30.06)	0.8
f2	9.20 (1.20, 11.75)	9.63 (1.98, 11.77)	0.9

Continuous variables with nonnormal data distribution are presented as medians (interquartile range).

d1: House dust mite; e5: Dog hair; m3: Mold; i6: Cockroach; f2: Milk.

*Significantly different between samples at the time of storage for sIgE allergens (p < 0.05).

**Table 2:** The correlation between serum levels of different allergens upon sample entry and sIgE measured.

sIgE	Correlation	Kappa
d1	rs = 0.967, P < 0.001	0.862
e5	rs = 0.786, P = 0.021	0.686
m3	rs = 0.917, P = 0.001	1.000
i6	rs = 1.000, P < 0.001	1.000

The Kappa statistic in the table represents a measure of agreement or consistency.

### The effect of storage time on the detection of allergen sIgE in serum samples

As shown in Table 3 and Figire 1B, the sIgE levels of samples stored for 10 years [9.42 kU/L (1.59, 60.85)] and samples stored for 5 years [10.04 kU/L (2.13, 33.65)] at the time of storage were higher than in subsequent testing [9.26 kU/L (1.73, 38.81) and 9.44 kU/L (1.90, 47.57)], respectively, but the difference was not statistically significant (P > 0.05). One sample of the sample stored for 10 years changed from a high positive level to a medium positive level, and one sample of the sample stored for 5 years changed from a medium positive level to a high positive level (Table S1). The sIgE level of samples stored for 3 years [4.75 kU/L (2.24, 57.28)] at the time of storage was lower than in subsequent testing [5.17 kU/L (1.56, 60.98)] (P > 0.05), and one sample changed from a medium positive level to a low positive level. The correlation between the sIgE levels at the time of storage and in subsequent testing was highest for samples stored for 10 years (*r*_*s*_ = 0.991, P < 0.001), followed by samples stored for 5 years (*r*_*s*_ = 0.964, P < 0.001), and the consistency was highest for samples stored for 10 years (Kappa = 0.884), followed by samples stored for 3 years (Kappa = 0.780, Table 4).

**Table 3:** Difference between sIgE levels measured in current samples and allergen serum samples stored for different periods of time.

sIgE	Pre-storage (kU/L)	Post-storage (kU/L)	P
G3	4.75 (2.24, 57.28)	5.17 (1.56, 60.98)	0.73
G5	10.04 (2.13, 33.65)	9.44 (1.90, 47.57)	1
G10	9.42 (1.59, 60.85)	9.26 (1.73, 38.81)	0.84

Continuous variables with nonnormal data distribution are presented as medians (interquartile range).

G3: samples stored for 3 years; G5: samples stored for 5 years; G10: samples stored for 10 years.

**Table 4:** The correlation between sIgE levels measured in current samples and allergen serum samples stored for different periods of time.

sIgE	Correlation	Kappa
G3	rs = 0.867, P < 0.001	0.78
G5	rs = 0.964, P < 0.001	0.73
G10	rs = 0.991, P < 0.001	0.884

### Influence of different positive levels on sIgE detection in serum samples

As shown in Table 5 and Fig. 1C, the sIgE levels of low positive samples [1.56 kU/L (1.32, 2.12) vs. 1.50 kU/L (0.59, 1.72)], medium positive samples [11.4 kU/L (6.32, 26.3) vs. 10.62 kU/L (5.47, 26.61)], and high positive samples [79.15 kU/L (65.6, 84.59) vs. 73.14 kU/L (60.45, 76.03)] at the time of storage were all higher than the results of subsequent testing, but the difference was not statistically significant (P > 0.05). Among them, there was 1 case of high positive samples dropping to medium positive level, 1 case of medium positive samples dropping to low positive level, and 1 case of medium positive samples increasing to high positive level. The correlation between the results of medium positive samples before and after testing (*r*_*s*_ = 0.994, P < 0.001) was the highest. The consistency in the positive levels of samples before and after testing was highest in low positive samples (Kappa = 0.810), followed by medium positive samples (Kappa = 0.707, Table 6).

**Table 5:** Difference between sIgE levels measured in current samples and allergen serum samples stored for different positive levels.

sIgE	Pre-storage (kU/L)	Post-storage (kU/L)	P
High level	79.15 (65.6, 84.59)	73.14 (60.45, 76.03)	0.24
Medium level	11.4 (6.32, 26.3)	10.62 (5.47, 26.61)	0.97
Low level	1.56 (1.32, 2.12)	1.50 (0.59, 1.72)	0.22

Continuous variables with nonnormal data distribution are presented as medians (interquartile range).

High level: samples of level 0–2; Medium level: level 3–4; Low level: level 5–6.

**Table 6:** The correlation between sIgE levels measured in current samples and allergen serum samples stored for different positive levels.

sIgE	Correlation	Kappa
High level	rs = 0.367, P = 0.332	–
Medium level	rs = 0.994, P < 0.001	0.707
Low level	rs = 0.448, P = 0.145	0.81

### Change rate of samples before and after testing with different positive levels and storage time for different types of allergens

The sample with the largest change rate before and after testing for house dust mite allergens (− 38.4%) was a level 5 positive sample, followed by a level 4 positive sample with a change rate of − 34.4% (Table S1). The sample with the smallest change rate (− 0.6%) was a level 3 positive sample. Interestingly, the change rate for dog hair allergens reached a maximum of − 99.1%, followed by − 97.9%. Both samples were stored for 3 years and had positive levels of 2 and 3, respectively. The change rate for tobacco mold allergens stored for 10 years was relatively high, at 38.1%, 20.6%, and − 32.0%, respectively. The maximum change rate for cockroach allergens was 40.2%, while the minimum was − 14.5%. Among them, there were three samples with a change rate < 17%, all of which were medium to low positive level samples. In addition, the maximum change rate for food allergen cow milk was only 13.2%, which had the smallest range of change rate among all allergens.

## Discussion

Biobanks, serving as essential resources for contemporary scientific research, underscore the paramount importance of long-term preservation and utilization of samples^12,15,24^. As a significant immunological marker, IgE plays a crucial role in numerous immunological studies. Whether it's used as a key biomarker for in vitro diagnosis of Type I hypersensitivity reactions, as a biological marker for asthma, or as an essential factor in autoimmunity, there are abundant reports attesting to its importance^25–27^. Simultaneously, advanced IgE measurement techniques offer us more options to assess sample variations through IgE concentration^28^. To investigate the influence of ultra-low temperature storage of serum samples on sIgE detection results, this study analyzed three factors, including different types of allergens, storage duration and different positive levels.

We found that some sIgE results from previous measurements were higher than those obtained in this study, while others were lower. Apart from systematic errors caused by the instrument, and accidental errors by the operator, long-term sample storage may cause solvent evaporation, resulting in an increase in sample concentration and leading to the former^29^. The latter may be due to the fact that the pH change during frozen storage of serum samples can affect the stability of proteins, leading to the formation of aggregates and precipitation^30^. At the same time, the activation of enzymes during the thawing process can affect the conformation and solubility of denatured proteins, promoting the aggregation and precipitation of proteins, resulting in a decrease in the solute content of the sample. This process can be effectively suppressed by adding glucose and polyacrylamide^31^. This indicates that changing the thawing conditions of the sample, adding appropriate buffers and protectants, and other methods can mitigate or reduce protein aggregation and precipitation during the thawing process. Moreover, studies have shown that the degradation of large peptides/proteins is minimized after several freeze–thaw cycles or long-term storage at − 80 °C, encouraging the use of valuable samples in archival biobanks for biomarker research^32^. Therefore, standardized sample preservation and management operations are crucial^17,33,34^.

Our results showed that differences in sIgE levels between samples of different types of allergens and storage durations had no statistically significant impact on the current test results, except for two samples with a positive difference of ± 1 level. Therefore, we could conclude that ultra-low temperature storage of serum samples does not significantly affect the stability of sIgE detection of different types of allergens and different storage durations. However, samples with high positive levels had a larger negative difference and absolute difference in sIgE levels between previous measurements and this study, than medium and low positive level samples. This may be due to the relatively small concentration span of the low-level region (0.35 kU/L–3.50 kU/L) and the large span of the high-level region (50.00 kU/L–≥ 100.00 kU/L) in the RAST level grading standard, resulting in larger differences in samples with higher positive levels.

In addition, our analysis of change rates found that most samples had low change rates, but each type of allergen had 1–2 samples with change rates exceeding 30%, without close correlation to storage duration or positive level. We considered that this might be due to (1) the phenomenon of "cold denaturation", where when serum samples are stored for a long time at temperatures far below the freezing point of water, the non-polar groups inside the antibody protein are exposed to water, causing changes in the intermolecular interactions of free energy and reducing the hydrophobic interaction of proteins and changes in tertiary structure^35,36^; (2) long-term freezing may lead to crystal formation of the buffering component in serum, resulting in changes in pH^37^; (3) different reagent batches used for the previous measurements may cause errors. (4) During the thawing process, ice recrystallization can affect the structural integrity of nearby proteins, leading to protein denaturation^38^; (5). We also discovered two samples with large changes in positive levels, both for dog hair allergens, with a change rate reaching 99%. This may be related to improper sample preservation and management during storage, highlighting the importance of standardized biorepository management^34,39^.

In summary, based on our findings, it appears that serum allergen-specific IgE (sIgE) demonstrates stability under ultra-low temperature storage conditions, suggesting the viability of establishing an allergy disease biorepository. Moreover, our preliminary observations lead us to hypothesize that the influence of ultra-low temperature storage on sIgE levels may be largely independent of allergen type. However, this hypothesis warrants further experimental investigation for confirmation. The establishment of such a biorepository holds substantial potential for enhancing research into disease mechanisms, improving clinical diagnosis and treatment approaches, fostering drug development, and overall, contributing significantly to the advancement of medical research services. This work lays a foundation for future studies and underscores the importance of robust biorepository infrastructure in allergy and immunology research.

### Supplementary Information

Supplementary Table S1.

## Data Availability

The datasets used and/or analysed during the current study available from the corresponding author on reasonable request.
